# Genetic Programs Between Steroid-Sensitive and Steroid-Insensitive Interstitial Lung Disease

**DOI:** 10.1007/s10753-023-01866-7

**Published:** 2023-08-10

**Authors:** Yanjiao Lu, Kun Tang, Shanshan Wang, Pengfei Gao, Zhen Tian, Meijia Wang, Jinkun Chen, Chengfeng Xiao, Jianping Zhao, Jungang Xie

**Affiliations:** 1 Department of Respiratory and Critical Care Medicine, National Clinical Research Center of Respiratory Disease, Tongji Hospital, Tongji Medical College, Huazhong University of Science and Technology, Wuhan, 430030 China; 2 Department of Pulmonary and Critical Care Medicine, The First Affiliated Hospital of Sun Yat-Sen University, No. 58 Zhongshan 2nd Road, Guangzhou, Guangdong, 510080 China; 3 Department of Respiratory and Critical Care Medicine, the First Affiliated Hospital of Henan University of Science and Technology, Luoyang, 471003 China; 4 Western University, 1151 Richmond Street, London, ON N6A 3K7 Canada; 5 Department of Biology, Queens University, Kingston, ON K7L 3N6 Canada

**Keywords:** Interstitial lung disease, Idiopathic pulmonary fibrosis, Cryptogenic organizing pneumonia, Corticosteroids, Inflammation

## Abstract

The effectiveness of corticosteroids (GCs) varies greatly in interstitial lung diseases (ILDs). In this study, we aimed to compare the gene expression profiles of patients with cryptogenic organizing pneumonia (COP), idiopathic pulmonary fibrosis (IPF), and non-specific interstitial pneumonia (NSIP) and identify the molecules and pathways responsible for GCs sensitivity in ILDs. Three datasets (GSE21411, GSE47460, and GSE32537) were selected. Differentially expressed genes (DEGs) among COP, IPF, NSIP, and healthy control (CTRL) groups were identified. Functional enrichment analysis and protein–protein interaction network analysis were performed to examine the potential functions of DEGs. There were 128 DEGs when COP versus CTRL, 257 DEGs when IPF versus CTRL, 205 DEGs when NSIP versus CTRL, and 270 DEGs when COP versus IPF. The DEGs in different ILDs groups were mainly enriched in the inflammatory response. Further pathway analysis showed that “interleukin (IL)-17 signaling pathway” (hsa04657) and “tumor necrosis factor (TNF) signaling pathway” were associated with different types of ILDs. A total of 10 genes associated with inflammatory response were identified as hub genes and their expression levels in the IPF group were higher than those in the COP group. Finally, we identified two GCs’ response-related differently expressed genes (*FOSL1* and *DDIT4*). Our bioinformatics analysis demonstrated that the inflammatory response played a pathogenic role in the progression of ILDs. We also illustrated that the inflammatory reaction was more severe in the IPF group compared to the COP group and identified two GCs’ response-related differently expressed genes (*FOSL1* and *DDIT4*) in ILDs.

**Supplementary Information:**

The online version contains supplementary material available at 10.1007/s10753-023-01866-7.

## BACKGROUND

Interstitial lung disease (ILD) describes a wide range of diffuse pulmonary disorders that affect lung parenchyma through inflammation and fibrosis [1]. Inflammation is associated with the initiation and progression of ILD [2, 3]. Corticosteroids (GCs) are classic anti-inflammatory drugs that have been widely used for the treatment of ILD [4, 5]. However, the therapeutic effects of GCs differ greatly depending on the subtype of ILD. Cryptogenic organizing pneumonia (COP) is a type of ILD with unknown causes. Patients with COP show well prognosis and response to systemic GC treatment [6], but the optimum dose and length of treatment are not known. Idiopathic pulmonary fibrosis (IPF), another type of ILD with unknown etiology, is characterized by a worse prognosis and poor response to GC treatment [7]. The preferred treatment of patients with non-specific interstitial pneumonia (NSIP) was immunosuppressants including GC [8]. Previous studies suggest that the diverse responses to GC treatment in ILD patients may result from the different expressions of GR-α and HDAC2, which play important roles in the sensitivity to GC [9, 10]. It is ascertained that different mechanisms underlying COP, IPF, and NSIP are responsible for altered GC sensitivity in these patients. Therefore, it is of great significance to compare gene expression profiles among patients with COP, IPF, and NSIP.

The widespread use of bioinformatics technology facilitates the exploration of the potential mechanisms of lung disease [11, 12]. Gene expression profiling based on bioinformatics analysis has been extensively performed to indicate gene expression patterns in human diseases, providing new hypotheses for disease occurrence and progression [13]. Although gene expression profiles of IPF and NSIP have already been reported [14, 15], it remains unclear whether there are differences when comparing the three different types ILDs at the transcriptional level.

In the current study, we aimed to compare the genetic programs in COP, IPF, and NSIP patients based on previously published microarray/high-throughput sequencing data from the Gene Expression Omnibus (GEO) datasets. Our findings may provide insights into the mechanisms of GC treatment or anti-inflammatory therapy in ILD.

## METHODS

### Database, Search Strategy, and Selection Criteria

Principal keywords including “idiopathic pulmonary fibrosis (IPF)” or “usual interstitial pneumonia (UIP),” “cryptogenic organizing pneumonia (COP),” and “non-specific interstitial pneumonia (NSIP)” were searched in the GEO repository (http://www.ncbi.nlm.nih.gov/projects/geo/) [16]. The database selection criteria were as follows: (1) original lung tissue mRNA expression datasets including control (CTRL), COP, IPF, and NSIP and (2) datasets consisting of upregulated and downregulated mRNAs. Datasets for cell lines/animal models/body fluids (such as serum and saliva) were excluded. Three expression profiling datasets (GSE21411—GPL570, GSE47460—GPL6480 and GPL14550, and GSE32537—GPL6244) were obtained from the GEO.

The total number of COP samples was eight. To compare the gene expression between patients with steroid-sensitive ILD and steroid-insensitive ILD, we selected sex-, age-, or pulmonary function test-matched CTRL, IPF patients, and NSIP patients from three datasets to perform a case–control study at a ratio of 1: 1 ~ 2: 1. Sixteen CTRL, sixteen IPF, and sixteen NSIP samples were included in the analysis.

### Expression Matrix, Probe Mapping, and Data Processing

Preprocessed expression matrix and probe annotation files of three GSE datasets were downloaded from the GEO repository. The probe annotation and its corresponding expression profile from different platforms were mapped to a common gene expression list as previously described [17]. Probes from different platforms were substituted by official gene symbols. Multiple expression measurements of one gene were replaced by the median expression value [18, 19]. All expression measurements were log2 transformed and then normalized using the cross-platform normalization (xpn) method by platforms.

### Data Repeatability Test and Analysis of Differentially Expressed Genes (DEGs)

Pearson’s correlation test was performed to evaluate intra-group repeatability. The correlations among all samples were visualized using heat maps. Principal component analysis (PCA) is commonly used for sample clustering, diversity analysis, gene expression analysis, and resequencing. The intra-group repeatability was assessed using sample clustering analysis. In the DEG analysis, genes with a *P*-value of ≤ 0.05 and a fold change (FC) of ≥ 2 were considered significantly differentially expressed. Volcano maps were generated to visualize DEGs in different groups. To investigate core genes’ response to GCs, 151 GCs response-related genes were extracted from the Gene Ontology and GO Annotations (https://www.ebi.ac.uk/QuickGO/), which provides detailed annotations of genes.

### Functional Annotations, KEGG Pathway, and Network Analysis of DEGs

DEGs were submitted to the online software Database for Annotation, Visualization, and Integrated Discovery (DAVID, version 6.8, http://david.abcc.ncifcrf.gov/) for typical batch annotation and Gene Ontology (GO) enrichment analysis. The Kyoto Encyclopedia of Genes and Genomes (KEGG) (https://www.kegg.jp/) pathway analysis was used to identify DEG-associated pathways. Also, DEGs were imported into the Search Tool for the Retrieval of Interacting Genes (STRING) (http://string-db.org) (version 10.5) for the construction of the protein–protein interaction (PPI) network. The Cytoscape (version 3.8.0) was used to select hub genes and visualize the PPI network.

## RESULTS

### Characteristics of Datasets and mRNA Selection

According to database selection criteria, three GSE datasets (GSE21411, GSE47460, and GSE32537) were retrieved for analysis. Considering that the total number of COP samples was eight, we selected sex-, age-, or pulmonary function test-matched CTRL, IPF patients, and NSIP patients from three datasets. The basic characteristics of COP patients, IPF patients, NSIP patients, and CTRL were summarized in Table 1. As GSE datasets were selected from different platforms with different probes, the common genes identified in all GSE datasets were merged. Finally, the total number of gene symbols was 6314 (Supplemental file1). To compare gene expression measurements from different platforms, the data were normalized using the xpn method and an integrated dataset was created.

**Table 1:** Clinical Characteristic of Samples

	CTRL (*n* = 16)	COP (*n* = 8)	IPF (*n* = 16)	NSIP (*n* = 16)
**Age**	58.5 (51.0–64.0)	53.0 (50.0–59.8)	57.5 (54.2–62.5)	57.0 (53.0–65.0)
**Gender**				
Female	7 (43.8%)	3 (37.5%)	7 (43.8%)	5 (31.3%)
Male	9 (56.3%)	5 (62.5%)	9 (56.3%)	11 (68.8%)
**FVC%**				
> 80%	5 (5/11)	3 (3/8)	4 (4/16)	3 (3/16)
50 ~ 80%	4 (4/11)	3 (3/8)	6 (6/16)	9 (9/16)
< 50%	2 (2/11)	2 (2/80	6 (6/16)	4 (4/16)
**DLCO%**				
> 40%	10 (10/11)	5 (5/6)	7 (7/12)	7 (7/12)
≤ 40%	1 (1/11)	1 (1/6)	5 (5/12)	5 (5/12)

*CTRL* healthy control, *COP* cryptogenic organizing pneumonia, *IPF* idiopathic pulmonary fibrosis, *NSIP* non-specific interstitial pneumonia, *FVC* forced vital capacity, *DLCO* diffusion capacity for carbon monoxide

Data are median (IQR) or *n*/*N* (%)

### Data Validation and Identification of DEGs

**Fig. 1:**
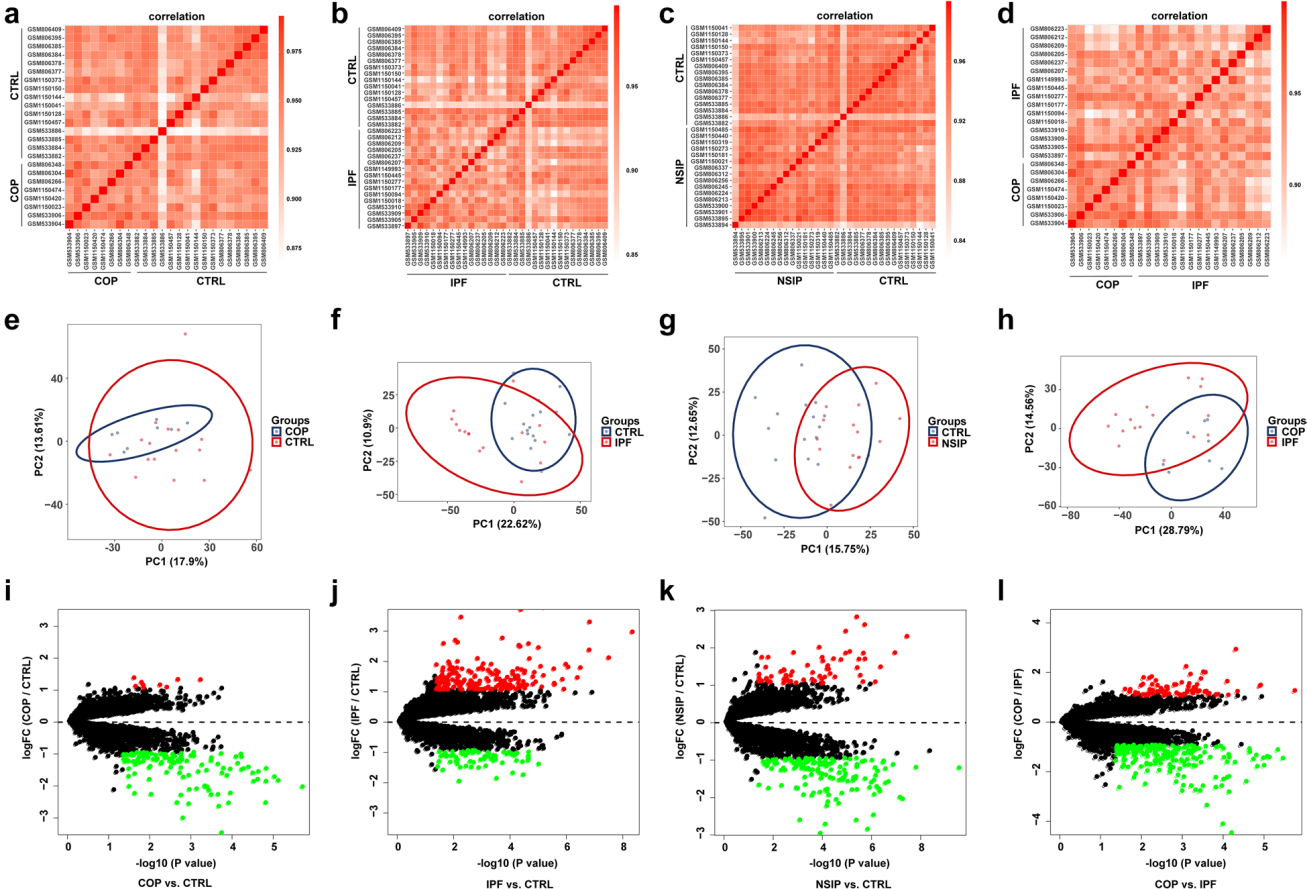
Comparisons of samples from COP, IPF, and CTRL groups. **a**–**d** Pearson’s correlation analysis of samples from different groups. The color indicates the intensity of the correlation. The greater the absolute value of the correlation coefficient, the stronger the correlation. 0 < correlation < 1: positive correlation; −1 < correlation < 0: negative correlation. **e**–**h** PCA of samples from different groups. In the PCA scatter diagram, the first principal component (PC1) and the second principal component (PC2) are used as *X*- and *Y*-axes, respectively. Each point represents a sample. The longer the distance between two samples, the greater the difference between them in gene expression patterns. **i**–**l** The volcano plot shows the differences between different groups. The fold change threshold was 2. Red dots: upregulated genes; green dots: downregulated genes.

To evaluate intra-group repeatability, Pearson’s correlation test and PCA were performed. The correlation analysis showed strong correlations in all groups (Fig. 1a–d, Supplemental Fig. 1a-b). The PCA suggested that the intra-group repeatability was acceptable. In addition, the distance between the samples from the same group was short, while the distance between the samples from different groups was long (Fig. 1e–h, Supplemental Fig. 1c-d).

**Fig. 2:**
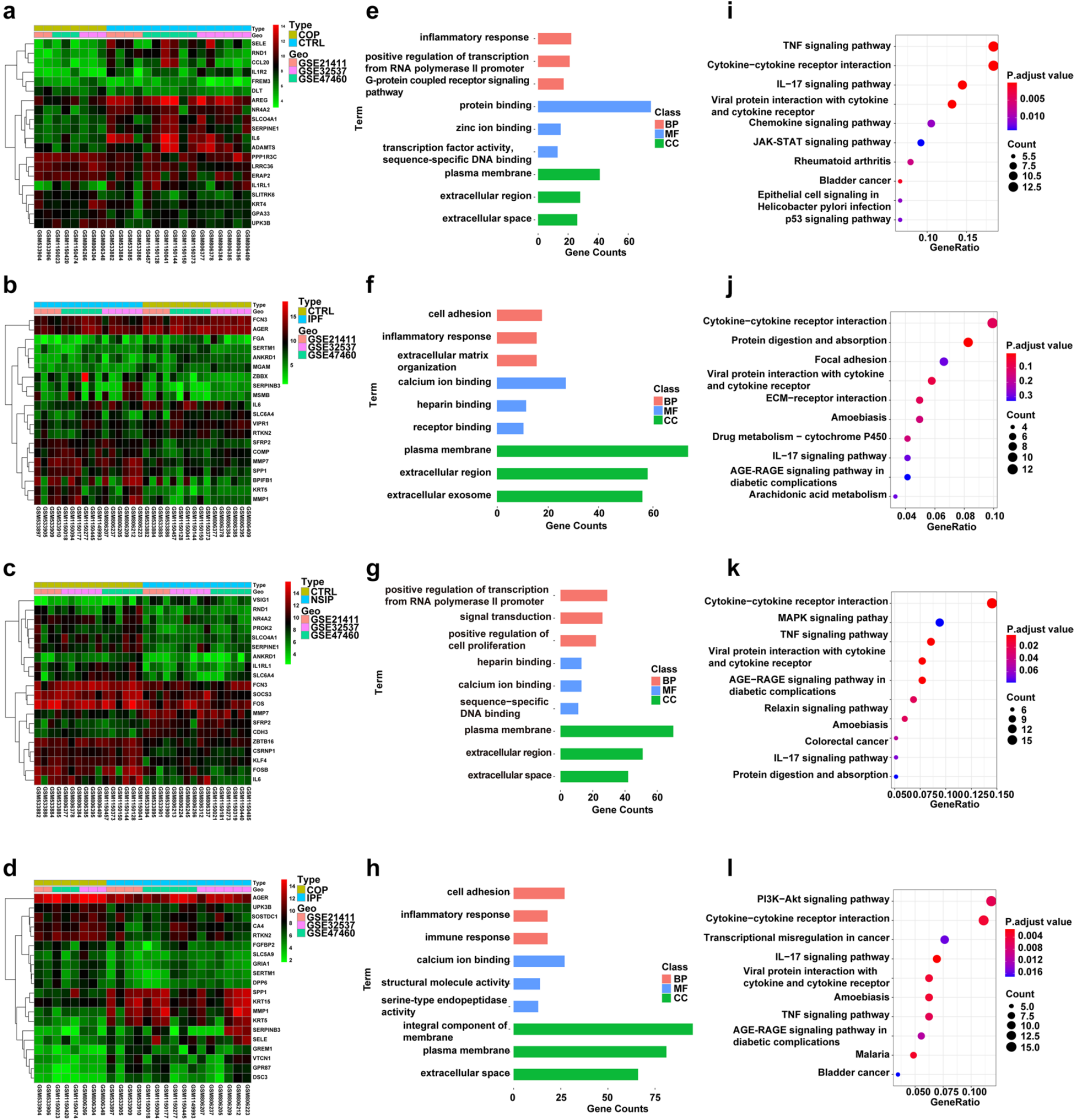
DEGs of different ILDs groups. **a**–**d** Hierarchical clustering of samples in different groups (COP vs. CTRL, IPF vs. CTRL, NSIP vs. CTRL, and COP vs. IPF) with the top 10 upregulated and the top 10 downregulated DEGs. **e**–**h** The GO enrichment analysis showed changes in the biological processes (BP), cellular components (CC), and molecular functions (MF) of DEGs in tissue samples from different ILDs groups. **i**–**l** The top 10 KEGG pathways analysis of DEGs that were identified. The scale of color legend represents the expression of the gene. CTRL *n* = 16, COP *n* = 8, IPF *n* = 16, and NSIP *n* = 16. DEGs, differentially expressed genes; KEGG, Kyoto Encyclopedia of Genes and Genomes; GO, Gene Ontology.

To further explore the mechanisms underlying disease development, we identified genes that were differentially expressed in different groups (*P*-value ≤ 0.05; FC ≥ 2). By comparing gene expression profiles between COP patients and CTRL, we identified 128 DEGs, among which 9 were upregulated and 119 genes were downregulated (Fig. 1I). By comparing IPF patients with CTRL, we found 257 DEGs, among which 72 genes were increased and 185 genes were decreased (Fig. 1J). We identified 205 DEGs when NSIP versus CTRL, with 63 up-regulated and 142 down-regulated (Fig. 1K). To investigate whether there were DEGs between steroid-sensitive and steroid-insensitive ILDs, we compared gene expression profiles between COP and IPF patients (Fig. 1L). The results showed that there were 270 DEGs (75 upregulated and 195 downregulated). What is more, the DEGs of COP versus NSIP and IPF versus NSIP were analyzed (Supplemental Fig. 1E-F). The top 20 DEGs of groups comparison were shown in Fig. 2A–D and Supplemental Fig. 2A-B. The detailed information of DEGs was provided in Supplemental file 2.

### Functional Annotation of DEGs by GO Enrichment Analysis and KEGG Pathway Analysis

Next, we performed GO enrichment analysis and KEGG pathway analysis to determine the function of DEGs (Fig. 2). The GO analysis revealed that DEGs were enriched in biological processes (BP), molecular function (MF), and cell components (CC). When comparing COP to CTRL, the most significantly-enriched BP terms were “inflammatory response” (GO:0006954), “positive regulation of transcription from RNA polymerase II promoter” (GO:0045944), and “G-protein coupled receptor signaling pathway” (GO:0007186). The most significantly enriched CC and MF terms were “plasma membrane” (GO:0005886) and “protein binding” (GO:0005515), respectively (Fig. 2E). The DEGs were enriched in numerous pathways, such as “cytokine-cytokine receptor interaction” (hsa04060), “TNF signaling pathway” (hsa04668), and “IL-17 signaling pathway” (hsa04657) (Fig. 2I). We also compared IPF with CTRL and the results of functional annotation were consistent with previous studies [20]. The most significantly-enriched BP terms were “cell adhesion” (GO:0007155), “inflammatory response” (GO:0006954), and “extracellular matrix organization” (GO:0030198) when IPF versus CTRL. While “plasma membrane” (GO:0005886) and “calcium ion binding” (GO:0005509) were separately enriched in CC and MF terms (Fig. 2F). The enriched KEGG pathway was “Cytokine-cytokine receptor interaction” (hsa04060), “Protein digestion and absorption” (hsa04974), and “Focal adhesion” (hsa04510) (Fig. 2J). When comparing NSIP to CTRL, the most significantly-enriched BP terms were “positive regulation of transcription from RNA polymerase II promoter” (GO:0045944) and “signal transduction” (GO:0007165). The most significantly enriched CC and MF terms were “plasma membrane” (GO:0005886) and “heparin-binding” (GO:0008201), respectively (Fig. 2G). “Cytokine-cytokine receptor interaction” (hsa04060), “MAPK signaling pathway” (hsa04010), and “TNF signaling pathway” (hsa04668) were the most enriched KEGG pathways (Fig. 2K). By comparing COP with IPF, we found that DEGs were highly enriched in BP, including “cell adhesion” (GO:0007155), “inflammatory response” (GO:0006954), and “immune response” (GO:0006955). The top GO terms of the CC and MF categories were “integral component of membrane” (GO:0016021) and “calcium ion binding” (GO:0005509), respectively (Fig. 2H). The notably enriched KEGG pathways were the “interleukin (IL)-17 signaling pathway” (hsa04657), “tumor necrosis factor (TNF) signaling pathway” (hsa04668), “malaria” (hsa05144), and “cytokine-cytokine receptor interaction” (hsa04060) (Fig. 2L). The GO and KEGG analyses were performed in DEGs of COP versus NSIP and IPF versus NSIP (Supplemental Fig. 2C-F). The detailed information of enrichment analysis was in Supplemental file 3.

**Fig. 3:**
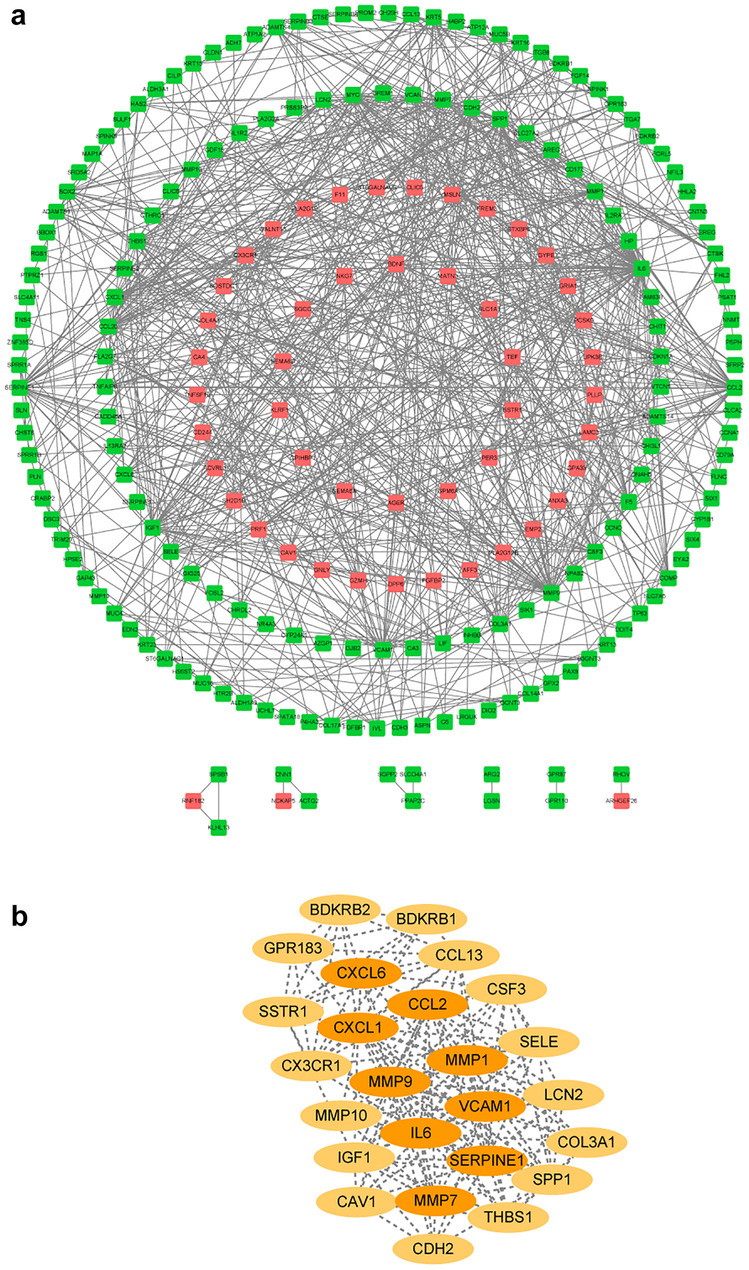
The PPI network of DEGs in COP vs. IPF. **a** The PPI network of DEGs. Edges represent protein–protein associations. Green circles: downregulation with a FC of > 2; red circles: upregulation with FC of > 2. **b** The top 25 hub genes from the PPI network were identified. PPI, protein–protein interaction.

The above results implied that ILD was closely related to inflammatory response, suggesting a correlation between GC sensitivity and inflammatory response in IIP. The pathway enrichment analysis of different groups revealed that the common pathways were the TNF signaling pathway and the IL-17 signaling pathway, which were the key components of the inflammatory network.

### PPI Network Construction, Hub Gene Selection, and Analysis

**Fig. 4:**
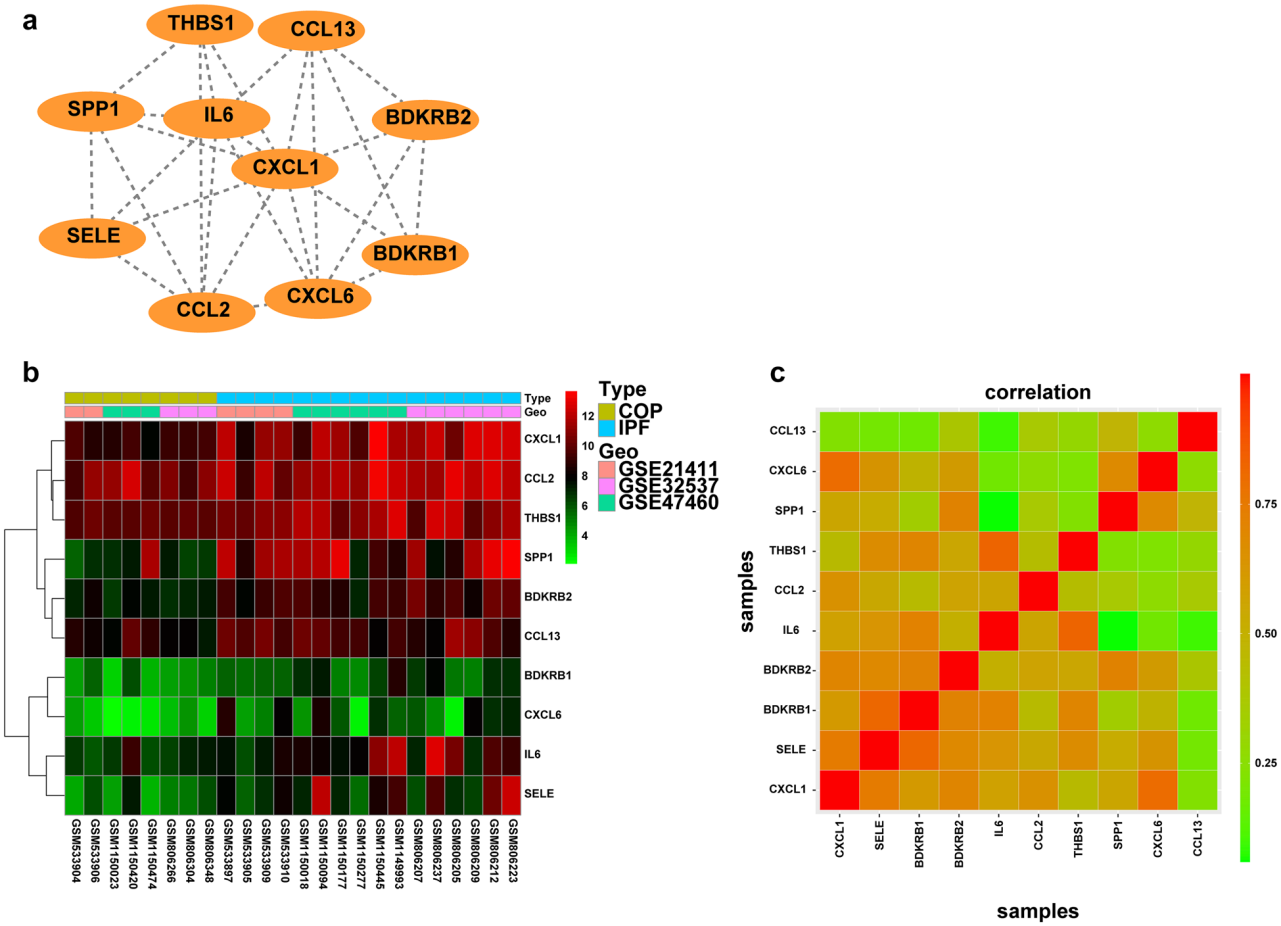
The hub genes associated with the inflammatory response in COP vs. IPF. **a** The 10 hub genes are associated with the inflammatory response. **b** Hierarchical clustering of samples in COP (*n* = 8) and IPF (*n* = 16) groups with 10 hub genes associated with the inflammatory response. **c** Pearson’s correlation analysis of 10 hub genes associated with the inflammatory response.

The molecular interaction networks were visualized by the STRING software based on various known evidence and the minimum interaction score was set to 0.40 (medium confidence). DEGs that were useful for distinguishing COP from IPF were submitted to the STRING and the gene network was generated (Fig. 3A). The PPI network contained 219 nodes and 748 edges. The hub genes in the PPI network were selected by the Cytoscape software (Fig. 3B). The top 25 hub genes were selected. Considering that inflammatory response played an important role in ILDs, we identified 10 hub genes that were also DEGs associated with inflammatory response, including *CXCL1, SELE, BDKRB1, BDKRB2, IL-6, CCL2, THBS1, SPP1, CXCL6*, and *CCL13* (Fig. 4A). All these genes were upregulated in IPF compared to COP (Fig. 4B) and the correlations among them were strong (Fig. 4C). Notably, SPP1 and SELE were also the top 20 DEGs when comparing COP with IPF.

### Core Gene Response to GCs in ILD

**Fig. 5:**
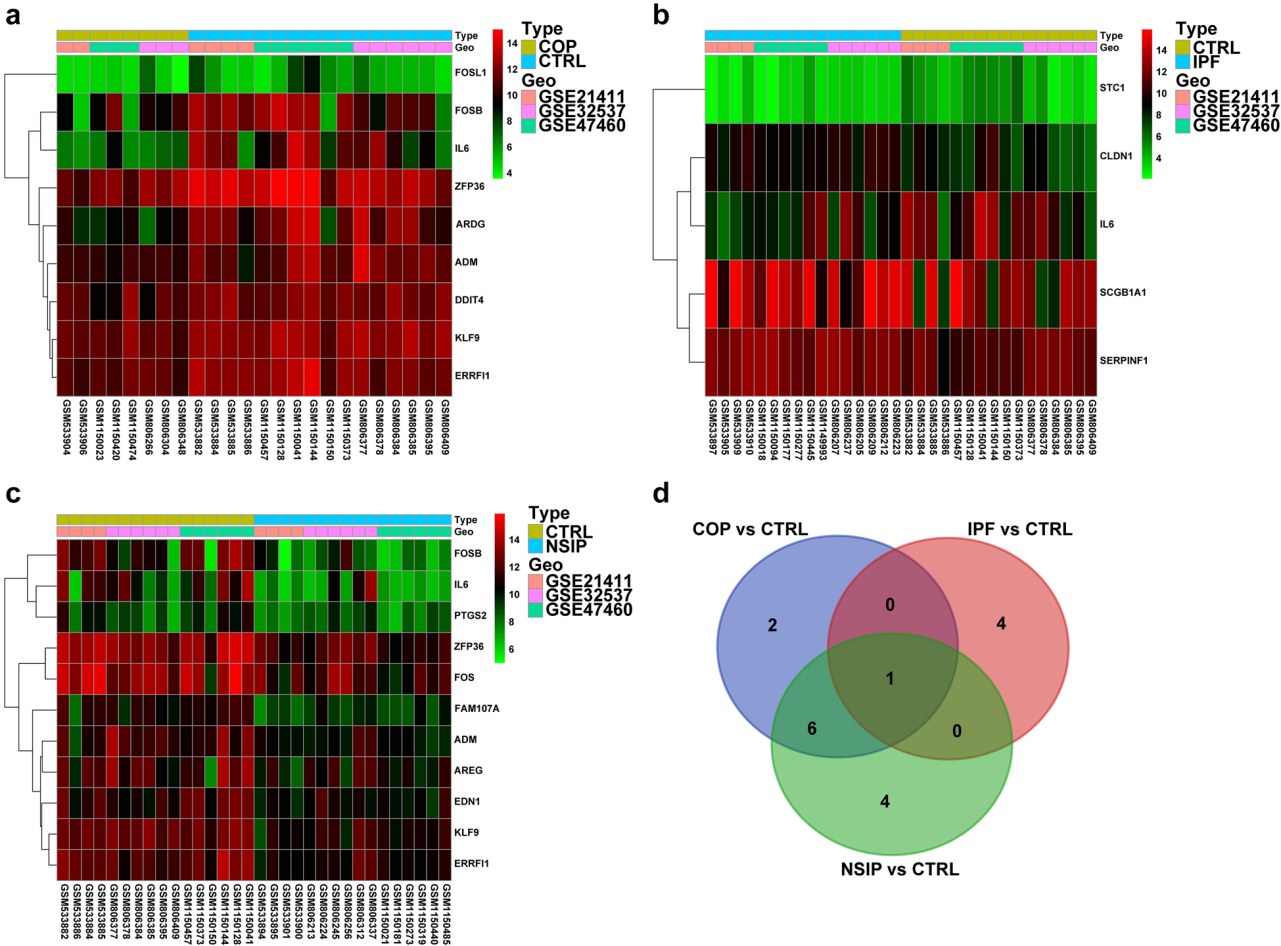
The GCs’ response-related differently expressed genes in ILDs. **a**–**c** The GCs’ response-related differently expressed genes in different groups (COP vs. CTRL, IPF vs. CTRL, and NSIP vs. CTRL). The scale of color legend represents the expression of the gene. **d** Venn diagram of the GCs’ response-related differently expressed genes in different groups.

To investigate core genes’ response to GCs in ILD, 151 GCs’ response-related genes were analyzed in different ILD groups. During the process of integrating the data, only 68 GCs’ response-related genes remained (Supplemental file 4). There were 9 GCs response-related differently expressed genes when comparing COP and CTRL (Fig. 5A), while 5 GCs response-related differently expressed genes in IPF versus CTRL (Fig. 5B) and 11 GCs response-related differently expressed genes in NSIP versus CTRL (Fig. 5C). Subsequently, to confirm core genes response to GCs in ILD, we overlapped the GCs’ response-related differently expressed genes in different group comparisons. As shown in the Venn diagram (Fig. 5D and Supplemental file 5), there were just two GCs response-related differently expressed genes (*FOSL1* and *DDIT4*) in the COP versus CTRL group compared to the other two groups. The two genes (*FOSL1* and *DDIT4)* might play important roles in a different response to GCs in ILDs.

## DISCUSSION

This analysis demonstrated differential gene expression patterns between different types of ILDs. By analyzing the DEGs, we found that IIP was closely related to the inflammatory response. Furthermore, the upregulation of 10 hub genes was associated with inflammatory response, indicating a more severe inflammatory reaction in IPF patients. The two genes (*FOSL1* and *DDIT4*) might play important roles in a different response to GCs in ILDs.

Global expression studies of lung tissues attempted to better elucidate the pathological mechanisms of ILDs. In our study, the analysis of three GSE datasets identified 128 DEGs between COP and CTRL. These genes were enriched in the inflammatory response, positive regulation of transcription from RNA polymerase II promoter, and G-protein coupled receptor signaling pathway. Previous studies have suggested the important role of these BP in the initiation and progression of COP [21–23]. When comparing IPF with CTRL, we confirmed 257 DEGs, which were enriched in cell adhesion and inflammatory response. We identified 205 DEGs when NSIP versus CTRL, significantly enriched in transcriptional regulation and signal transduction. The inflammatory response was also play important role in NSIP progression [8]. Although lung tissues affected by COP and IPF displayed similar signatures, 270 DEGs were identified, among which 75 were upregulated and 195 were downregulated in COP patients relative to the IPF group. We also found that the DEGs were mainly enriched in the inflammatory response and cell adhesion, which was consistent with previous studies showing that inflammatory reaction played a pivotal role in the pathogenesis of ILD (21–23).

GC administration is a classic treatment, but the effect of GCs differs depending on the type of ILDs. For instance, GC treatment is ineffective for IPF patients but effective for the COP population (24, 25). By comparing gene expression patterns in different ILDs, we attempted to identify the molecules and pathways essential for GC sensitivity.

Although inflammatory response-related pathways were enriched in both COP and IPF [22, 24, 25], the inflammatory response was the one of the most significantly enriched BP terms when comparing COP with IPF. Moreover, the KEGG analysis showed that the TNF and IL-17 signaling pathways were significantly different between the COP and IPF groups. A previous study reported that Chemokine C–C motif ligand 2 (CCL18), which was enriched in the inflammatory response, was significantly differentially expressed in COP patients compared to the IPF group [26], indicating a vital role of inflammatory response in COP and IPF. Consistent with our results, previous findings have confirmed that genes related to the TNF signaling pathway are abnormally expressed in COP and IPF [22, 27]. It has also been reported that GC treatment suppressed the secretion of IL-6 and TNF-α [28].

We have identified 10 hub genes related to the inflammatory response. Most of them were the downstream signal molecules of GC-related pathways [29–31]. IL-6 is a classical systemic inflammatory cytokine that can be inhibited by GCs in a variety of diseases [32, 33]. Chemokine C–C motif ligand 2 (CCL2), also known as monocyte chemoattractant protein-1 (MCP-1), is associated with the efficacy of GC treatment [29]. ThrombospondinNA1 (THBS1) was also involved in the response to GC treatment [34]. Furthermore, the 10 hub genes that were associated with inflammatory response were also significantly upregulated in IPF compared with the COP group, indicating that inflammatory reaction was more severe in IPF patients, which was consistent with the study by Bin et al. [10]

Although several anti-inflammatory drugs, such as anti-TNF-α drugs and GCs, failed to meet their primary endpoints or even exhibited detrimental effects [35, 36], new drugs targeting inflammatory response are currently under phase II and III clinical trials [37]. The different expressions of GR-α and HDAC2 in ILD patients might lead to varied responses to GC treatment (8, 9). However, in our study, there was no significant difference in the expression of HDAC2 among different types ILDs. Also, the expression of GR-α in different groups was not shown here due to the absence during the merging process. Therefore, we speculated that other signaling pathways might be involved in GC sensitivity in ILDs.

To investigate core genes’ response to GCs in ILDs, we analyzed GC response-related differently expressed genes in different ILDs. GC response-related differently expressed genes were distinct among different ILDs compared to CTRL. IL-6, a classic inflammatory cytokine, participated in to response of GC treatment in three ILDs. As COP showed well prognosis and response to systemic GC treatment, the two genes (FOSL1 and DDIT4) were only differently expressed in COP compared to IPF and NSIP. FOSL1 mediated the inhibition of GCs/GCs receptor signal [38], while the role of FOSL1 in the GC sensitivity of ILDs remains unknown. DDIT4 regulated cell growth and survival via inhibition of the activity of autophagy via mammalian target of rapamycin complex 1 (mTORC1) [39]. DDIT4 was reported to be regulated by GCs in brain function [40]. Whether DDIT4 could participate in the GC sensitivity of ILD via regulating autophagy still deserved research.

There are some limitations to this study. Firstly, the sample size of COP was small. Secondly, the effects of epigenetic modifications were not assessed, such as histone modifications, DNA methylation, and non-coding RNAs. Thirdly, the cross-platform analysis did not include all genes. Fourth, differences in the extent of tissue infiltration by inflammatory cells or inflammatory cell makeup in biopsy specimens between NSIP, COP, and IPF samples cannot be excluded and may contribute to different gene signatures detected in this analysis. Fifth, the history of medicine, another cofounding of this survey, was not reported in this study. Studies with larger sample size and detailed information on medicine taking are needed to further elucidate the mechanisms of GC sensitivity in ILDs.

## CONCLUSIONS

In summary, our bioinformatics analysis in different ILDs demonstrated for the first time that the gene expression profiles were significantly different, suggesting a pathogenic role of the inflammatory response during the progression of ILD. We also showed that the inflammatory reaction in IPF patients was more severe than that in the COP group, indicating the involvement of inflammatory response in disease occurrence and progression. Furthermore, the two genes (FOSL1 and DDIT4) might play important roles in a different response to GCs in ILDs.

### Supplementary Information

Below is the link to the electronic supplementary material.

Supplementary figure 1 (TIF 80457 KB)

Supplementary figure 2 (TIF 99839 KB)

Supplementary file 1 (XLSX 86 KB)

Supplementary file 2 (XLSX 105 KB)

Supplementary file 3 (XLSX 215 KB)

Supplementary file 4 (XLSX 11 KB)

Supplementary file 5 (XLSX 9 KB)

## Data Availability

All data generated or analyzed during this study are included in this published article (and its supplementary information files).

## Abbreviations

ILD: Interstitial lung disease
GCs: Corticosteroids
COP: Cryptogenic organizing pneumonia
IPF: Idiopathic pulmonary fibrosis
NSIP: Non-specific interstitial pneumonia
CTRL: Control
GEO: Gene Expression Omnibus
PCA: Principal component analysis
DEGs: Differentially expressed genes
GO: Gene Ontology
KEGG: Kyoto Encyclopedia of Genes and Genomes
BP: Biological processes
MF: Molecular function
CC: Cell components
